# Analysis of nutrition clinical studies involving children in the Middle East and globally

**DOI:** 10.4155/fsoa-2018-0067

**Published:** 2018-07-31

**Authors:** Fatih Ozdener, Feza Kirbiyik, Ali Evrim Dogan

**Affiliations:** 1 Department of Pharmacology, School of Medicine, Bahcesehir University, Yenisahra-Kadıkoy Istanbul, 34734, Turkey; 2 Medical Department, Nutricia Advanced Medical Nutrition, 34330, Turkey

**Keywords:** children, clinical studies, Middle East, nutrition, nutrition industry

## Abstract

**Aim:**

To assess pediatric clinical nutrition research by analyzing clinical studies in the Middle East (ME) and globally.

**Methods:**

Using ClinicalTrials.gov, the numbers of clinical studies in the ME and globally were analyzed.

**Results:**

The majority of clinical nutrition trials are in North America and Europe. The ME accounts for 4% of all nutrition trials. The majority of pediatric nutrition studies in the ME are in the later phases or are observational and/or epidemiological studies with a focus on poor nutrition or nutrition disorders. Industry funding in the ME is mostly by regional or local companies; few major global companies are involved.

**Conclusion:**

The ME is not well represented in clinical nutrition studies involving children. Effort should be expended to rectify this.

## Lay abstract

Nutritional disorders have become an important worldwide disease burden during the last several decades. The development of effective therapies for nutritional disorders requires clinical trials in the affected populations. Our results showed that although the Middle East currently has the highest proportion of children worldwide, it is not well represented in clinical trials on nutritional studies in children. Furthermore, there is suboptimal funding from the multinational nutrition industry. This analysis indicates that there is a reasonable rationale for inclusion of the Middle East in nutritional clinical research in children, and that there is an untapped opportunity for the expansion of clinical research in this region.

Nutritional disorders and undernourishment are important health problems globally, although the disorder spectrum and underlying root causes can vary considerably according to the level of development of the country [1]. While most of the underdeveloped world continues to suffer from a spectrum of disorders due to malnutrition, the developed world is dealing with an epidemic of obesity caused by an unhealthy diet [2].

Prevention, diagnosis and treatment of nutritional problems arising in childhood are important as these conditions can lead to profound and irreversible developmental problems [3]. The higher birth rates, proportions of children, and prevalence of undernourishment in the underdeveloped world increase susceptibility to nutritional problems. The Middle East (ME), which has experienced rapid population growth in the past century, has the highest proportion of children worldwide (about one-third of the population is <15 years of age) [4,5]. Due to poor healthcare, but not a low income, the child malnutrition rate in the ME is approximately 17% [6,7].

Although the causes of poor nutrition and its consequences have been investigated in various geographical regions [8], the degrees of involvement of these regions in clinical nutrition research are unclear. The ME, with its high proportion of children in the population and high prevalence of malnutrition, is suitable for conducting clinical nutrition studies. In this study, we assessed pediatric clinical nutrition studies in the ME.

## Methods

### ClinicalTrials.gov database

ClinicalTrials.gov is a database of privately and publicly funded clinical studies conducted worldwide, and is the world's largest publicly available clinical trials registry and results database [9]. Currently, it encompasses more than 260,000 research studies in more than 200 countries. Although it is by far the largest database for ongoing and completed clinical trials, it does not include all studies. It is thought that the dataset in the registry is more complete for studies conducted by multinational companies [10]. The World Medical Association's Declaration of Helsinki states that every research study involving human subjects must be registered in a publicly accessible database before recruitment of the first subject (including human nutrition intervention studies) [11,12]. Using ClinicalTrials.gov, we identified ongoing clinical nutrition trials in the ME according to region, country, age group, funder and phase. Countries were assigned to regions according to the ClinicalTrials.gov database, and the searches were conducted during the fourth quarter of 2017.

### Database search

ClinicalTrials.gov was searched using the advanced search function. All medical conditions containing words relevant to ‘nutrition’ were included in the search terms (for example, poor nutrition and nutritional stunting). The age group from birth to 17 years old was selected as the eligibility criterion for inclusion, and adults (18–64) and older adult (65+) populations were excluded where necessary. Studies for both male and female sexes were included. All study types, interventional, observational (including patient registries) and expanded access studies were analyzed. All studies with or without results were included regardless of the recruitment status. Additional analysis criteria were funder type (industry vs all others) and study phase (early, Phases I through IV). Cyprus, Iran, Iraq, Israel, Jordan, Kuwait, Lebanon, Oman, Qatar, Syrian, Turkey, United Arab Emirates and Yemen with at least one study listed in ClinicalTrials.gov, were categorized in the ME region and were included in the search for the ME.

#### Study limitations

Although ClinicalTrials.gov was the first online registry for clinical trials and is the largest and by far most widely used, it is not the only registry for clinical trials. Therefore, it is expected that not all nutritional studies, but the vast majority of such studies are registered at ClinicalTrials.gov. In addition, some countries do not strictly require clinical trial registration, but strongly encourage it. Therefore, it is possible that some nutritional studies are not listed in any of the registries. Finally, some trials, although registered, could have been reported in an imprecise or incomplete manner and therefore may not appear under the appropriate category in the registry for analysis.

## Results

**Figure 1.**
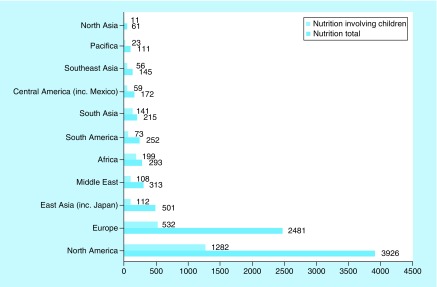
**Comparison of regional distribution of clinical nutrition studies in children and all age groups.** ClinicalTrials.gov was searched for clinical studies with the keyword ‘nutrition’ entered in the ‘condition or disease’ field. The search was initially conducted globally and involved all age groups categorized according to geographical region. Subsequently, the same search was conducted for children, categorized according to the geographical region and compared with the results for all age groups.

**Figure 2.**
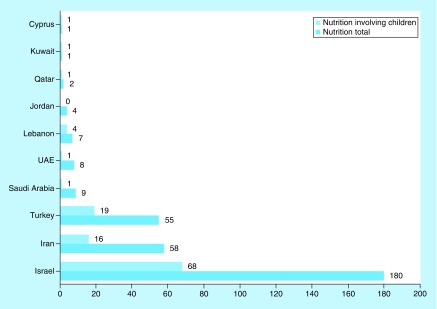
**Comparison of clinical nutrition studies in the Middle East categorized by country.** ClinicalTrials.gov was searched for clinical studies with the keyword ‘nutrition’ entered in the ‘condition or disease’ field. The search was conducted for all age groups and subsequently restricted to children. Studies that appeared under the ME region were subsequently categorized according to country and the numbers of studies in children were compared with that of all age groups. ME: Middle East.

First, we investigated the regional distribution of clinical nutrition trials. Around three quarters of all ongoing nutrition clinical trials are in North America and Europe (76%, 6407 of 8470) and one quarter in the rest of the world (24%, 2063 of 8470) (Figure 1). The ME accounted for 4% (313 of 8470) of all nutrition trials, ranking fourth after North America, Europe and Asia. In the ME, the majority (90%) of ongoing trials are in Israel, Turkey and Iran (Figure 2).

Second, we investigated the distribution of clinical nutrition studies involving children. Despite the high proportion of children in the ME, the proportion of clinical trials was low (4%), similar to total nutrition clinical trials in the ME (4%) (Figure 1). Furthermore, the majority of ongoing clinical nutrition studies involving children are in Israel, Turkey and Iran (92%) (Figure 2).

**Figure 3.**
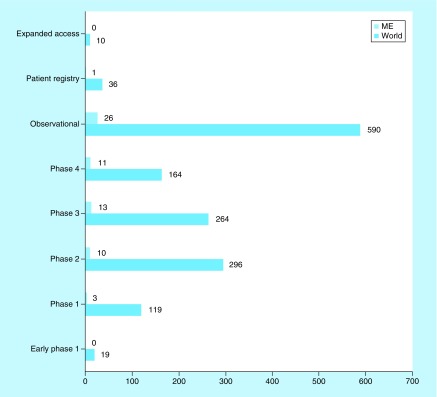
**Phases and types of clinical nutrition studies involving children.** ClinicalTrials.gov was searched for clinical studies with the keyword ‘nutrition’ entered in the ‘condition or disease’ field. The search was initially conducted globally and involved the pediatric age group categorized according to phase or type of study. Subsequently, the same search was conducted for the ME and the numbers were compared with the global search. ME: Middle East.

Early phase interventional studies are conducted more frequently in developed regions; developing countries are generally included in later developmental phases. Only one Phase I trial is being conducted in the ME; the majority are at later phases or are observational or epidemiological (Figure 3). Furthermore, there is only one registry study in the ME, and no expanded access study.

**Figure 4.**
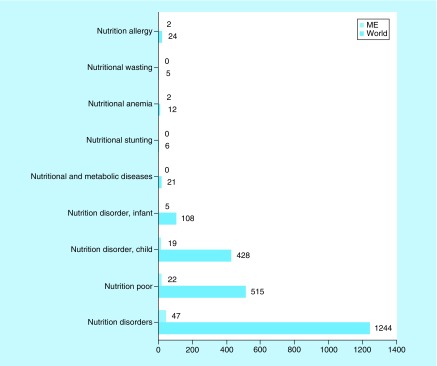
**Medical conditions or diseases targeted by nutrition studies involving children in the Middle East and globally.** ClinicalTrials.gov was searched repeatedly for clinical studies with keywords entered each time for a different medical condition or diseases in the ‘condition or disease’ field. The search was initially conducted globally and involved the pediatric age group. Subsequently, the same search was conducted for the ME and the numbers were compared with global search. ME: Middle East.

We also investigated the medical conditions targeted by the ongoing clinical nutrition trials in the ME. Poor nutrition or nutrition disorder studies predominated (97 and 96%, respectively) in the ME, followed by metabolic disorders, anemia and allergy studies (Figure 4).

**Figure 5.**
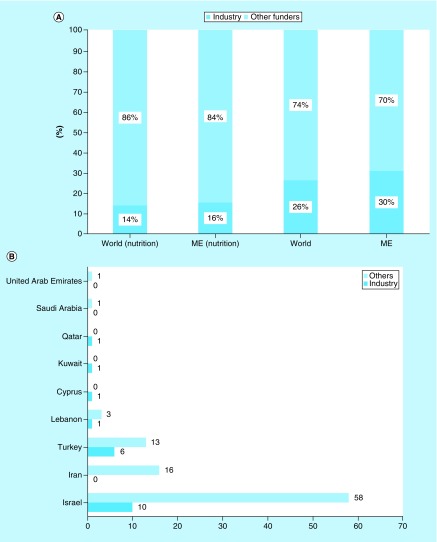
**Sources of funding for clinical nutrition studies.** **(A)** Sources of funding for clinical nutrition studies involving children. ClinicalTrials.gov was searched for clinical studies with the keyword ‘nutrition’ entered in the ‘condition or disease’ field. The search was initially conducted globally and involved the pediatric age group categorized according to funder of the study (industry or all other sources). Subsequently, the same search was conducted for the ME, and the numbers were compared with the results of the global search. **(B)** Sources of funding for clinical nutrition studies involving children in the ME by country. ClinicalTrials.gov was searched for clinical studies with the keyword ‘nutrition’ entered in the ‘condition or disease’ field. The search was conducted for the ME and involved the pediatric age group categorized by country according to funder of the study (industry or all other sources). ME: Middle East.

**Figure 6.**
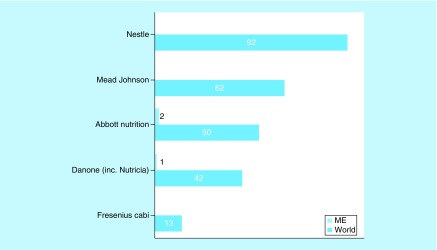
**Major multinational nutrition industry funding of nutrition studies involving children.** ClinicalTrials.gov was searched for clinical studies with the name of a major multinational nutrition company entered in the ‘sponsor’ field each time. The search was initially conducted globally and involved the pediatric age group categorized according to sponsor. Subsequently, the same search was conducted for the ME and the numbers were compared with the results of the global search. ME: Middle East.

Next, we analyzed the sources of funding (industry, academic or state) for clinical nutrition studies. Industry is sponsoring fewer pediatric nutrition studies relative to total nutrition studies both globally (14 vs 26%) and in the ME (16 vs 31%) (Figure 5A). The highest proportion of industry-funded studies was in Turkey (31%), followed by Israel (15%), but none of the studies in Iran were industry-funded (Figure 5B). One clinical nutrition study involving children is being conducted in each of Cyprus, Kuwait and Qatar, all are funded by the nutrition industry. Globally, the major industrial funders of clinical nutrition trials were Nestle, Mead Johnson, Abbott Nutrition and Danone Nutricia (Figure 6). However, most funding is from small regional or local companies; Abbott Nutrition and Danone Nutricia are currently sponsoring just two and one studies in the ME, respectively.

## Discussion

The feasibility of a particular country or region for inclusion in a clinical study has well-defined clinical, regulatory, technical and operational components [13]. The availability of clinical epidemiological data regarding the prevalence of the disease condition for the intended clinical study is a primary determinant of the outcome of most feasibility studies. The prevalence rates of disease conditions associated with inadequate nutrition are high and continue to grow in countries of the MENA regions, with high prevalence rates of malnutrition, micronutrient deficiencies, obesity and diseases closely related to food consumption, dietary patterns and lifestyles [14].

The ME has the youngest population in the world and a high incidence of nutritional disorders, mostly due to poor health awareness [15]. We investigated the number of clinical nutrition studies involving children in the ME. Around three-quarters of clinical nutrition trials were in North America and Europe, and only one-quarter in the rest of the world. The ME ranks fourth, with 4% of all clinical trials. Regarding clinical nutrition studies involving children, the ME ranks sixth (4%). Previously, Misik analyzed the geographical distribution of clinical trials (CTs) conducted in all therapeutic areas and relative contributions of emerging markets, including the Middle East and North Africa (MENA) region [16]. Misik reported that the majority of CT sites (78%) and patients (65%) were from developed countries with emerging regions accounting for only 22% of the CT sites and almost 35% of patients. Furthermore, MENA (excluding Israel), only contributes 1% of the emerging market share. The analysis projected an increase in percentage of global CTs in MENA paralleling an increase in sales market and pointed to MENA as the region with strongest growth potential of its CT market. In contrast to our analysis based on direct CT numbers, this conclusion was based on CT sites and patient numbers, which were roughly correlated with CT numbers.

Selection of a country for inclusion in clinical studies is dependent on the patient pool as well as the Good Clinical Practice (GCP) laws and regulations, the number of feasible sites, and the availability of qualified and experienced investigators [17]. It has been proposed that widespread adoption of the International Conference on Harmonization (ICH)-GCP guidelines and stronger intellectual property protection in developing countries could contribute to the globalization of clinical research [18]. This is consistent with our observation that GCP standards and investigator qualifications are closely correlated with the number of studies for some countries in the ME. For example, Israel has long been involved in clinical trials and the number of ongoing clinical studies in Israel is comparable to that in Western European countries [17]. The presence of experienced investigators that have already adopted ICH-GCP makes Israel an attractive country for inclusion in clinical trials. Israel currently ranks first in the ME in the number of clinical nutrition studies involving children. Turkey is another country that has been able to increase the adoption of ICH-GCP as a result of well-organized GCP courses directed toward potential clinical investigators. As a result, Turkey has become one of the few countries (along with China and South Korea) that has increased the number of clinical trials performed within its borders significantly in recent years. Turkey currently ranks second in the ME in the number of clinical nutrition studies involving children. Increasing clinical research through rapid adoption of guidelines and regulations can be a feasible approach for other countries in the ME that currently run only a few such studies.

Our results showed that, globally, the largest number of studies are at Phase II, followed by Phase III, Phase IV and Phase I. This is similar to the situation regarding other therapeutic areas, as Phase II is a bottleneck in product development [19,20]. Few Phase I trials are currently ongoing in the ME, but compared with global figures, proportionally larger numbers of Phase III and Phase IV trials are underway in this region. This is because new therapeutics are first tested in the country in which they are developed, and Phase III trials require large numbers of participants worldwide. Other possible reasons for the globalization of late-phase clinical trials were proposed to be substantial cost savings by conducting trials in developing countries, shortening the timeline for clinical testing and overcoming the increasingly bureaucratic and expensive regulatory environment in many wealthy countries [18]. As expected, we found large numbers of observational/epidemiological clinical nutrition studies involving children after product registration, patient registries and expanded  access programs globally. In contrast, there was only one patient registry, and no expanded access studies in the ME. Expanded access programs are important as they provide alternatives for patients who have exhausted their treatment options [21].

Next, we investigated the medical condition targeted by the ongoing clinical studies in the ME. Despite the low socioeconomic status and poor nutritional awareness in certain parts of the region, the proportions of studies on poor nutrition and nutritional disorders were identical in the ME and globally (both, 97 and 96%, respectively). Studies on metabolic disorders, anemia and allergy comprised a minor proportion of those ongoing in the ME. Furthermore, there was no difference between the ME and the rest of the world in terms of the proportion of trials on poor nutrition and the rates for both were considerably high (23 and 22%, respectively). This result was expected as there was marked emphasis on studies on poor nutrition due to an increased awareness of their importance. It has recently been reported that poor nutrition is not only detrimental for the early stages of life but also for midchildhood (5–9 years) and adolescence (10–14 years) [3]. Malnutrition increases mortality in midchildhood and limits growth in adolescence.

According to Day and Jackson, the nutrition industry plays a fundamental role in transforming clinical research into new products and is a key partner for driving clinical nutrition studies [22]. Therefore, we analyzed the sources of funding for clinical nutrition studies involving children in the ME. There was no significant difference in the proportion of industry funding of clinical nutrition studies involving adults or children between the ME and the rest of the world. The industry is sponsoring fewer pediatric nutrition studies relative to total nutrition studies both globally (14 vs 26%, respectively) and in the ME (16 vs 31%, respectively). This may explain the unexpectedly low proportion of pediatric nutrition studies in the ME, despite its high proportion of children. Of the ongoing clinical nutrition trials involving children in Turkey and Israel, a moderate proportion was funded by industry (31 and 15%, respectively); however, none of the ongoing trials in Iran was funded by the industry. This difference warrants further investigation. In contrast, the one ongoing clinical nutrition study in each of Cyprus, Kuwait and Qatar were funded by industry, signaling the future interest of the nutrition industry in these countries. Nestle, Mead Johnson, Abbott Nutrition and Danone Nutricia are the major funders of ongoing clinical nutrition trials among children in the ME. However, in the ME the majority of funding is from smaller regional or local companies; Abbott Nutrition and Danone Nutricia are sponsoring two and one studies, respectively.

In summary, the majority of ongoing clinical nutrition studies involving children are in North America and Western Europe; far fewer are being conducted in the ME, despite its high proportion of children. In the ME, the majority of ongoing clinical nutrition studies involving adults and children are in Israel, Turkey and Iran. Ongoing studies in the ME are predominantly at later phases or are observational and/or epidemiological studies of nutrition disorders or poor nutrition. The level of nutrition-industry funding for clinical nutrition studies involving children is lower than that for studies involving adults both in the ME and globally. Few major nutrition companies are funding clinical studies in the ME.

## Conclusion

Due to the high proportion of children, high birth rate and high prevalence of poor nutritional status, countries in the ME are suitable for conducting clinical nutrition studies in children. Realization of this potential would have positive ethical, medical and marketing implications for the region, and the nutrition industry will be likely to play an important role in achieving this through funding activities.

## Future perspective

The clinical development arena is highly competitive when it comes to timely recruitment of subjects for clinical studies. Designing a sensible clinical study with tight timelines requires careful consideration of the worldwide distribution of patients with the disease of interest and the number of competing studies for the same population in a specific region. A high proportion of children combined with high prevalence of poor nutritional status make the ME a suitable place for conducting clinical nutrition research in children. Fast adoption of the ICH-GCP guidelines, increasing the number of qualified investigators and improving the operational infrastructure will all be equally important in the realization of this potential. The nutrition industry is likely to play an important role in achieving this through its funding. This analysis is an example of how we can best utilize the clinical trial registries to identify opportunities, thereby optimizing clinical studies and increasing the efficiency of clinical development. We envision that such analyses will be valuable in the future and will potentially be of benefit to both nutritional and pharmaceutical industries.

**Summary points**

- Nutritional disorders and undernourishment are becoming important health problems globally, necessitating the development of effective therapeutic measures supported by clinical research.
- Our analysis showed that the majority of nutritional clinical research is conducted in North America and Europe, with all remaining regions, including the Middle East (ME), accounting for about one quarter of all studies.
- Despite its relatively high pediatric population and high prevalence of malnutrition, the ME is not currently well represented in clinical studies for the pediatric population.
- The condition spectrum of investigations in the ME is strongly predominated by poor nutrition or nutrition disorder studies.
- For the ME region, industrial funding relies strongly on regional or local companies, with only limited participation of international nutrition companies.
- We concluded that there is an important opportunity for expansion of nutritional clinical research in the pediatric population in the ME, which can possibly be driven by the multinational nutrition industry.
